# Autocrine and paracrine STIP1 signaling promote osteolytic bone metastasis in renal cell carcinoma

**DOI:** 10.18632/oncotarget.15222

**Published:** 2017-02-09

**Authors:** Jiang Wang, Hongbo You, Jun Qi, Caihong Yang, Ye Ren, Hao Cheng

**Affiliations:** ^1^ Department of Orthopedics, Tongji Hospital, Tongji Medical College, Huazhong University of Science and Technology, Wuhan 430030, P.R.China

**Keywords:** RCC bone metastasis, stress-induced phosphoprotein 1(STIP1), STIP1-ALK2-SMAD1/5 signaling, STIP1-prion-ERK1/2 signaling, tumor microenvironment

## Abstract

Bone metastases are responsible for some of the most devastating complications of renal cell carcinoma (RCC). However, pro-metastatic factors leading to the highly osteolytic characteristics of RCC bone metastasis have barely been explored. We previously developed novel bone-seeking RCC cell lines by the *in vivo* selection strategy and performed a comparative proteome analysis on their total cell lysate. Here, we focused on STIP1 (stress-induced phosphoprotein 1), the high up-regulated protein in the bone-seeking cells, and explored its clinical relevance and functions in RCC bone metastasis. We observed high levels of both intracellular and extracellular STIP1 protein in bone metastatic tissue samples. Elevated STIP1 mRNA in the primary RCC tumors remarkably correlated with worse clinical outcomes. Furthermore, both human recombinant STIP1 protein and anti-STIP1 neutralizing antibody were used in the functional studies. We found that 1) STIP1 protein on the extracellular surface of tumor cells promoted the proliferation and migration/invasion of RCC tumor cells through the autocrine STIP1-ALK2-SMAD1/5 pathway; and 2) STIP1 protein secreted into the extracellular tumor stromal area, promoted the differentiation of osteoclasts through the paracrine STIP1-PrPc-ERK1/2 pathway. Increased cathepsin K (CTSK), the key enzyme secreted by osteoclasts to degrade collagen and other matrix proteins during bone resorption was further detected in the differentiated osteoclasts. These results provide evidence of the great potential of STIP1 as a novel biomarker and therapeutic target in RCC bone metastasis.

## INTRODUCTION

RCC is one of the ten most common cancers in adults, and clear cell RCC represents 85% of these cases [1]. The number of RCC diagnoses has increased by ∼40% over the past three decades, but the overall 5-year overall survival rates have improved very little in that time (www.cancer.gov). Almost one-third of patients with newly diagnosed RCC have metastases at initial presentation, and another third will see their disease metastasize within ten years. The 5-year survival rate for metastatic RCC is less than 10% [2]. RCC bone metastasis, the second most common site for RCC following lung [3, 4], is particularly aggressive and destructive, as it causes more skeletal-related events (SREs) than bone metastases from other cancers, especially pathological fracture, spinal cord and nerve root compression, and hypercalcemia [5, 6]. Furthermore, while new targeted therapies have significantly increased patient survival, bone has become a sanctuary site in RCC [7]. The understanding of bone metastasis in RCC is, therefore, becoming increasingly important to facilitate the development of preventive and therapeutic strategies.

Early onset and highly osteolytic are the two distinctive characteristics of RCC bone metastasis compared with common bone metastasis from other cancers, i.e., breast and prostate cancers [8]. Bone metastasis from RCC develops even before diagnosis and is often accompanied with comorbid metastases in other organs in a relatively short period. This course suggests that aggressive pro-metastatic factors intrinsically exist in the tumor cells to foster metastatic colonization. Studies on breast and prostate cancer indicate that the mechanisms for tumor growth in bone are complex involving tumor stimulation to osteoclasts and osteoblasts as well as their responses in the bone microenvironment [9]. In particular, tumor cells have been shown to produce many factors to stimulate osteoclastic osteolysis, including interleukin (IL)-6, IL-8, IL-11, parathyroid hormone-related protein (PTHrP), and receptor activator of nuclear factor-κB ligand (RANKL). As a consequence of osteoclastic resorption, growth factors released from the bone matrix further enrich the local milieu of tumor cells. This vicious cycle between tumor cell and bone accelerates cancer progression. However, these molecular insights from the studies on breast and prostate cancers are obviously not specific to RCC, and thus, they do not explain the praecox osteolytic feature of bone metastasis in RCC. Furthermore, bone metastasis in RCC is much less investigated than those in breast and prostate cancer, and the tumor pro-metastatic factors have barely been explored.

It is well-recognized that certain tumor cells (‘seed’) have specific affinity for the milieu of certain organs (‘soil’) [10]. Based on this rationale, we used the previously reported *in vivo* selection strategy and developed novel bone-seeking clear cell RCC cell lines (OS-RC-2-BM5 and ACHN-BM5) [11, 12]. To characterize the molecular factors that endow RCC tumor cells with the ability to survive and cause lytic bone metastases, we performed a comprehensive proteome analysis on the bone-seeking RCC cells in comparing with their corresponding parental cells [12]. We focused on the protein level analysis rather than mRNA, because 1) we reasoned that the interplay between tumor cells and bone cells are largely mediated by secretory proteins, and 2) the protein analysis is more indicative to reveal corresponding cellular alterations than changes in mRNA levels.

## RESULTS

### Elevated expression and secretion of STIP1 in RCC bone metastasis

**Figure 1:**
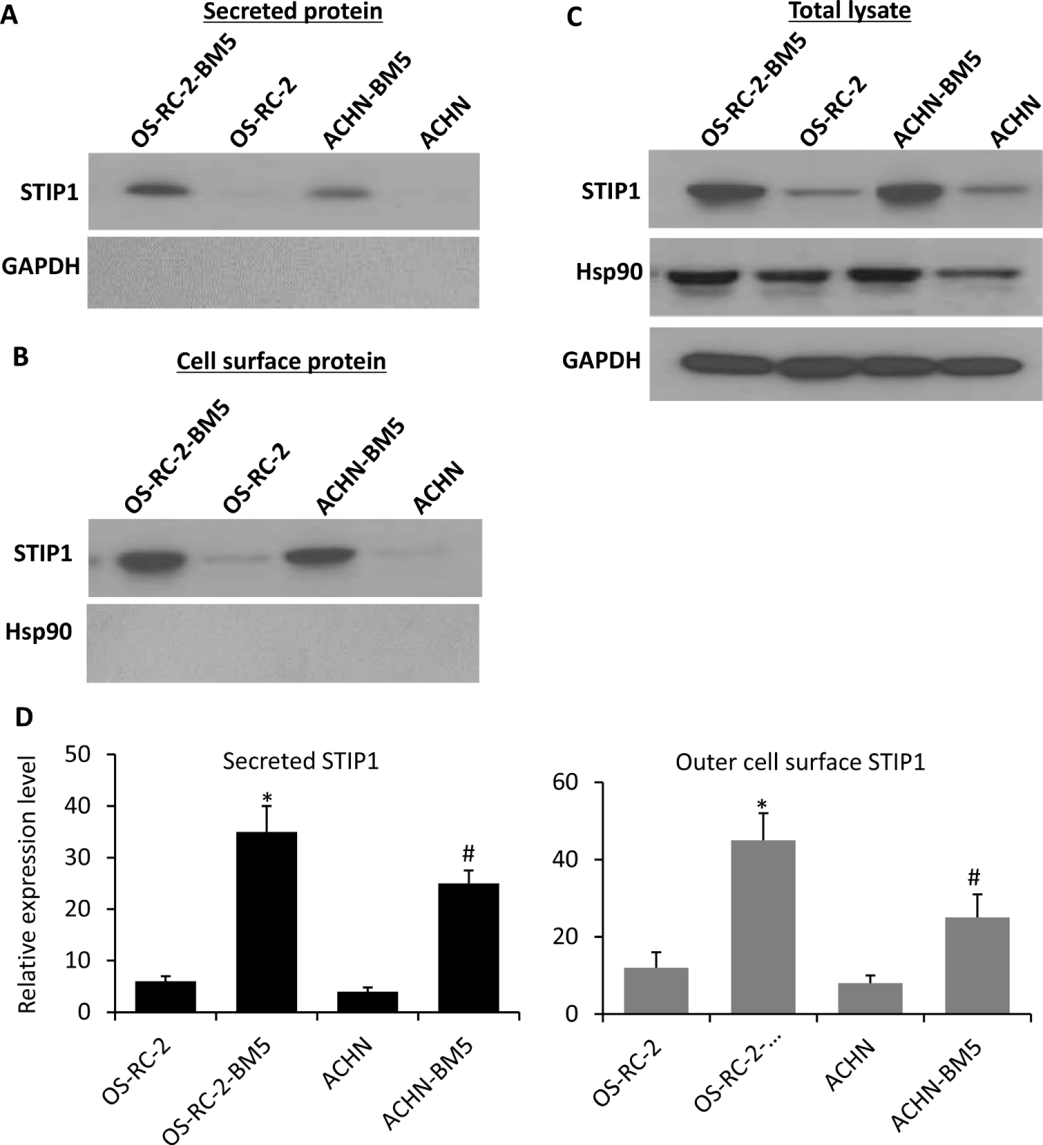
STIP1 in RCC tumor cells transported to cell membrane for secretion into culture medium (**A**) After 24-h culture of the RCC tumor cells, secreted STIP1 in the culture medium supernatant was detected, while the intracellular protein GAPDH was not detected in the culture medium supernatant indicating no leakage of intracellular components into the culture media. (**B**–**C**). STIP1 was detected in the purified cell surface protein, while no trace of HSP90 was identified, while HSP90 was detected in the total cell lysates (C). (**D**) Quantification of STIP1 protein in the culture medium supernatant as secreted STIP1 (left panel), and in the purified cell surface protein as outer cell surface STIP1 (right panel). Experiments were triplicated, and mean ± SD was presented. *p < 0.05, *vs* OS-RC-2; ^#^*p <* 0.05, *vs* ACHN. In all panels, western blot images have been cropped to show the protein of interest, and all blots were performed under the same experimental conditions.

In our previous proteome analysis of the total cellular protein from RCC tumor cells, STIP1 protein was found to be 4-fold more abundant in the bone-seeking cells than in the control parental cells [12], and STIP1 was observed to be translocated to the cell surface and secreted out of cells [13, 14], which may mediate extracellular tumor-niche interactions. To further define the subcellular location of the increased STIP1 protein, and study whether tumor cells actively secrete STIP1 protein, we conducted two experiments and found First, After culturing RCC tumor cells were cultured in serum-free medium for 24 h, we observed that the culture media clearly contained STIP1 but no intracellular GAPDH protein (Figure 1A). Second, we isolated cell membrane proteins using the Pierce Cell Surface Protein Isolation kit and detected STIP1 on the extracellular surface of the plasma membrane of the OS-RC-2-BM5 and ACHN-BM5 cells (Figure 1B). We used HSP90 as a marker for possible contamination with intracellular proteins. There was no trace of HSP90 in the isolated cell surface proteins (Figure 1B), but HSP90 was abundant in the total cell lysates (Figure 1C). Furthermore, both experiments confirmed that the bone-seeking cells secreted more STIP1 or contained more STIP1 protein on the outer cell membrane than the parental cells (Figure 1D).

**Figure 2:**
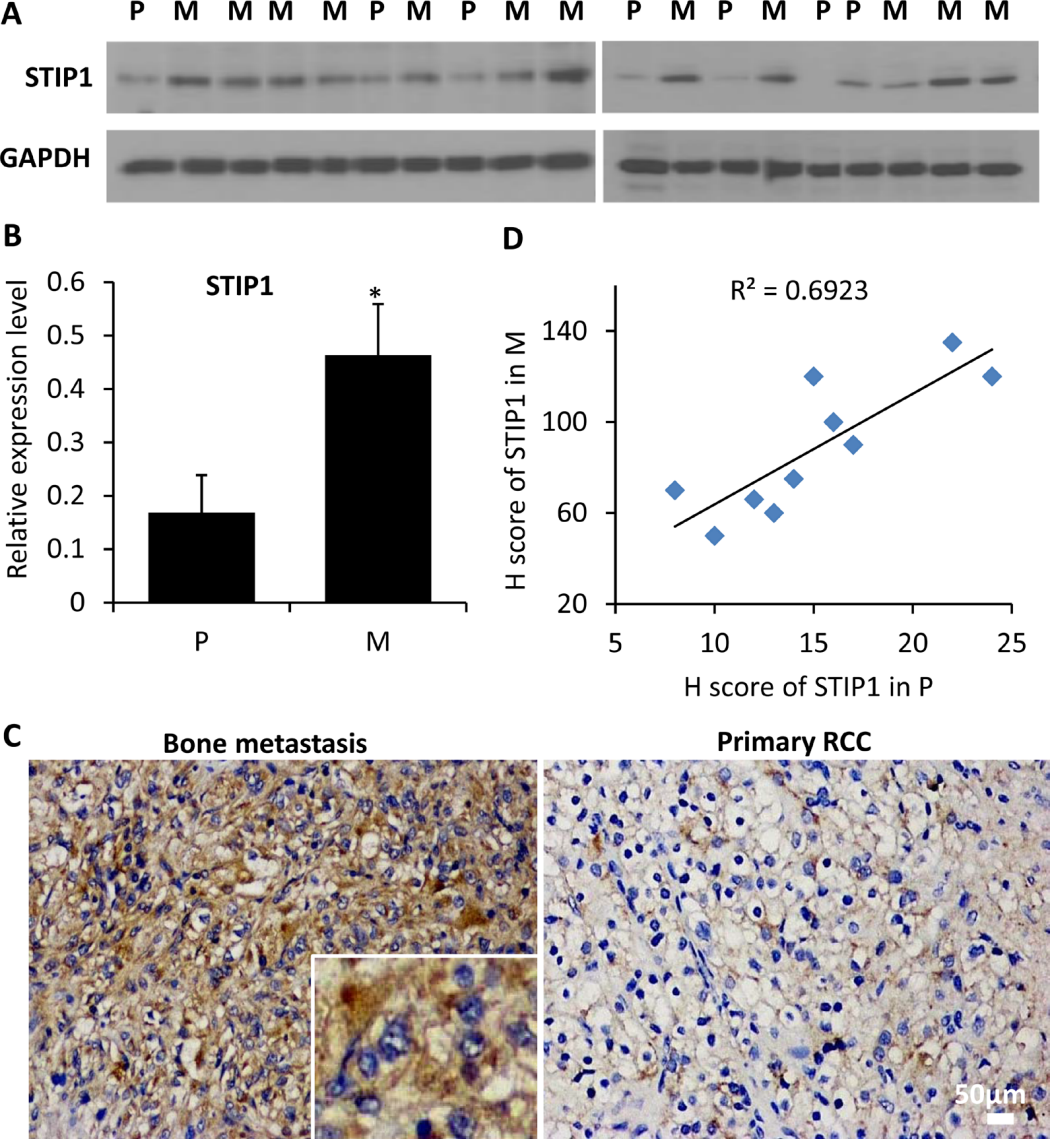
STIP1 protein expression in clinical samples (**A**) STIP1 protein was examined in 19 protein specimens from primary RCC tumors (P, *n* = 7) and bone metastatic samples (M, *n* = 12). Proteins were electrophoresed in two 10% SDS-PAGE gels, and were subsequently transferred to two PVDF membranes (10 and 9 specimens for gels 1and 2, respectively). (**B**) The intensity of STIP1 in each lane was normalized with the intensity of GAPDH. *p < 0.05. Experiments were duplicated, and western blot images shown have been cropped to show the protein of interest, and all blots were performed under the same experimental conditions. (**C**) Representative immunohistochemistry staining of STIP1 in primary RCC and bone metastasis tumors. Both intracellular and extracellular STIP1 immunoreactivity was examined as shown in the inset. Images were taken under 20× objective. Scale bar: 50 μm. (**D**) Correlation between the H scores of STIP1 in primary RCC and bone metastasis tumors of the 10 pairs of matched samples. R^2^ = 0.6923.

To explore the clinical relevance of our findings, we quantified the expression of STIP1 protein in tissue biopsy or surgically removed fresh primary RCC samples (*n* = 7) and bone metastasis samples (*n* = 12) by Western blot analysis. Nineteen protein specimens were electrophoresed in two gels. Statistically significant differences in STIP1 levels were identified between primary RCC and bone metastasis (Figure 2A, 2B). Furthermore, we examined the immunoreactivity of the STIP1 protein in 10 pairs of matched primary RCC and bone metastasis paraffin-embedded tissues (Figure 2C, 2D), and found a strong correlation of the high STIP1 expression in primary tumors with bone metastatic tumors (the Pearson's correlation coefficient is 0.69). Both intracellular and extracellular STIP1 immuno-reactivities were detected (Figure 2C), which further confirmed the secretion of STIP1 into the tumor stromal area.

**Figure 3:**
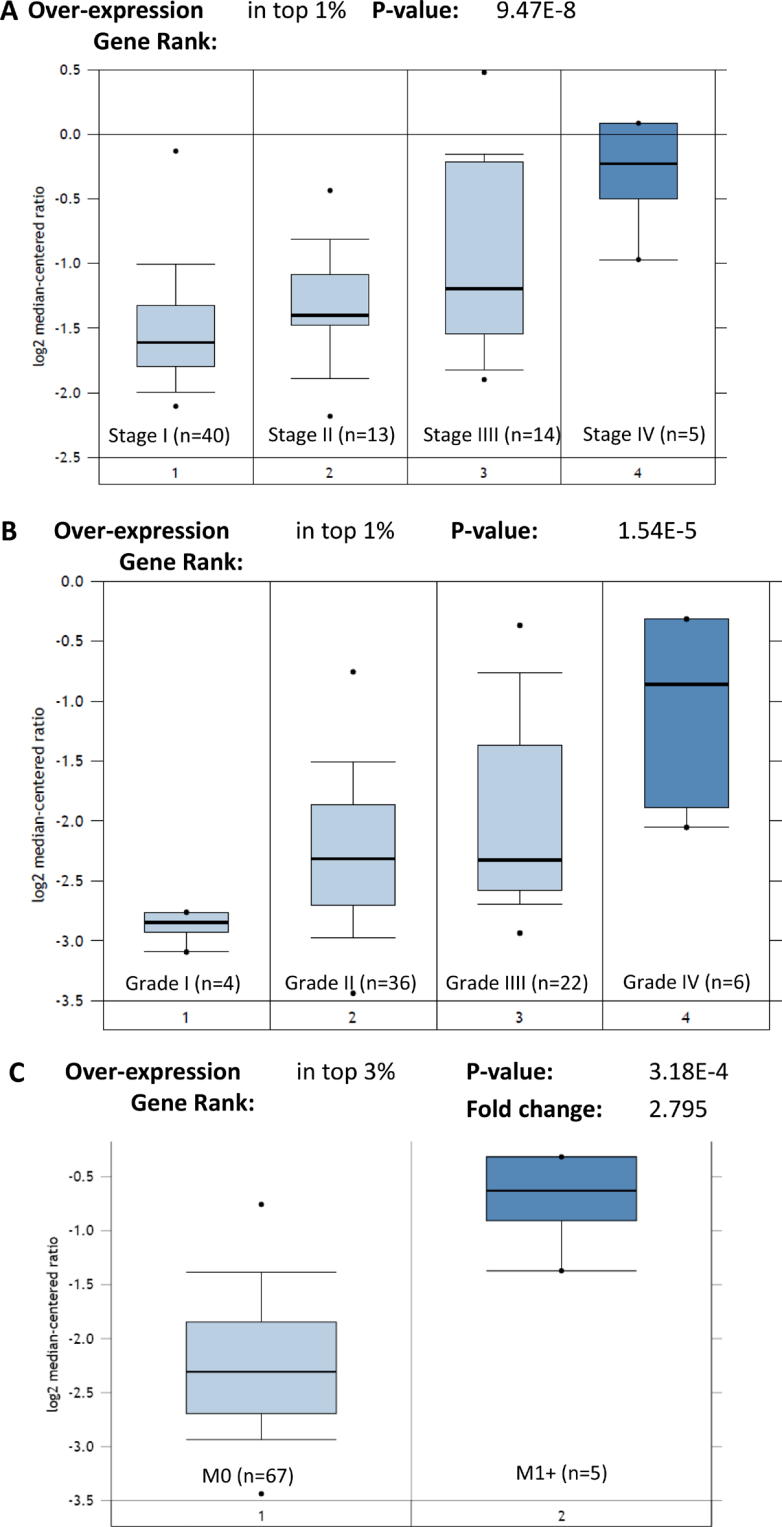
Expression of *STIP1* mRNA in TCGA Renal cohort (**A**) *STIP1* mRNA expressed highly in the advanced stage RCC tumors. (**B**) *STIP1* mRNA expressed highly in the high grades RCC tumors. (**C**) *STIP1* mRNA expressed highly in the metastatic RCC tumors (M1+). Overexpression gene rank and *P value* were generated by the Oncomine algorithms. Fold change was log 2 based.

STIP1 is the gene coding for the STIP1 protein. We also identified that the high expression of *STIP1* mRNA remarkably correlated with advanced stages (*P* = 9.47E-8), high grades (*P* = 1.54E-5), and metastasis status (*P* = 3.18E-4) in the TCGA Renal dataset (*n* = 88) (Figure 3). These results indicate that the elevated transcription of *STIP1* mRNA directly leads to the high expression of STIP1 protein. Altogether, these results suggest STIP1 as a clinically relevant factor in mediating RCC tumor development, and motivated us to explore its specific functions in bone metastasis.

### STIP1 promotes tumor cell proliferation, migration, and invasion through the autocrine STIP1-ALK2-SMAD1/5 pathway

**Figure 4:**
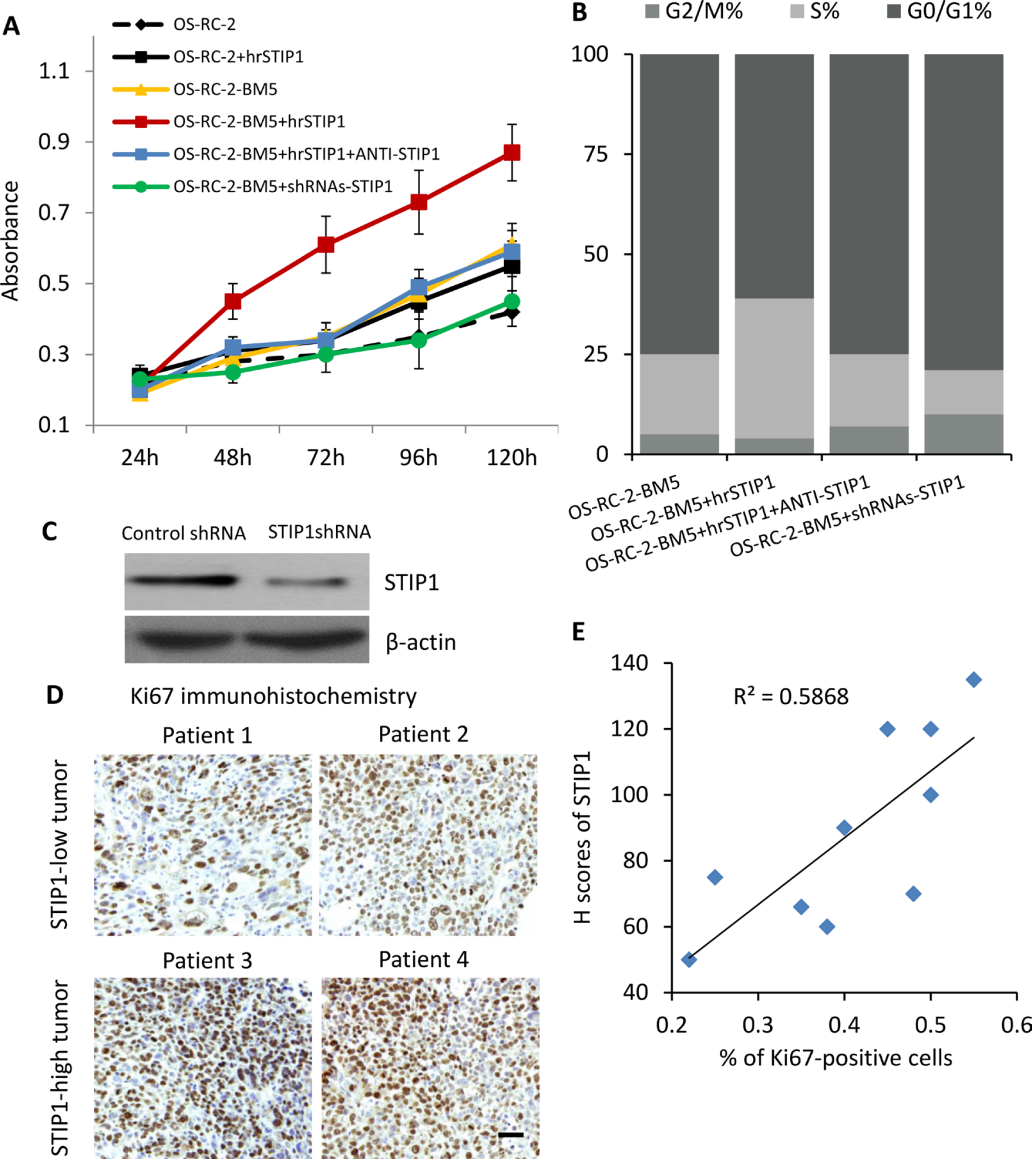
STIP1 promotes tumor proliferation (**A**) Proliferation of OS-RC-BM5 cells under indicated treatment. (**B**) Cell cycle analysis of OS-RC-BM5 cells under indicated treatment. (**C**) shRNA knockdown of STIP1 in OS-RC-BM5 cells. (**D**) Representative images of Ki67 immunoreactivity in the bone metastasis tumors. Images were taken under 20× objective. Scale bar: 50 μm. (**E**) Correlation between the H scores of STIP1 and percentage of Ki67-positive cells in the same bone metastasis tumors (*n* =). R^2^ = 0.5868.

Under the normal monolayer culture condition, both MTT assay and flow cytometry cell cycle analysis revealed that the bone-seeking cells proliferated faster than the parental cells [11, 12]. To test whether STIP1 modulates the proliferation of tumor cells, we added human recombinant STIP1 (hrSTIP1) to the culture media. Relative to the vehicle-treated cells, the hrSTIP1-treated OS-RC-2-BM5 cells showed about 2-fold increase of the proliferation rate at 72 hours and nearly double of the cells entered S phase (Figure 4A, 4B). Furthermore, co-treatment with the anti-STIP1 neutralizing antibody abrogated these effects (Figure 4A, 4B), indicating the specific pro-proliferation role of STIP1 in the tumor cells. shRNAs specific to STIP1 were used to knock down the STIP1 protein expression in OS-RC-2-BM5 cells (Figure 4C), and reduced cell proliferation was observed (Figure 4A, 4B).

Ki67 is a nuclear protein that is associated with cell proliferation. We used immunohistochemistry for endogenous Ki67 as an indicator to evaluate the *in vivo* tumor cell proliferation in the previously mentioned bone metastasis patient tissue samples. The results also demonstrated that increased levels of STIP1 were positively correlated with increased numbers of Ki67-positive tumor cells (the Pearson's correlation coefficient is 0.59, Figure 4D, 4E). Although it is just a correlation analysis between STIP1 and Ki67, these results provided *in vivo* evidence on STIP1 promoting tumor cell proliferation.

**Figure 5:**
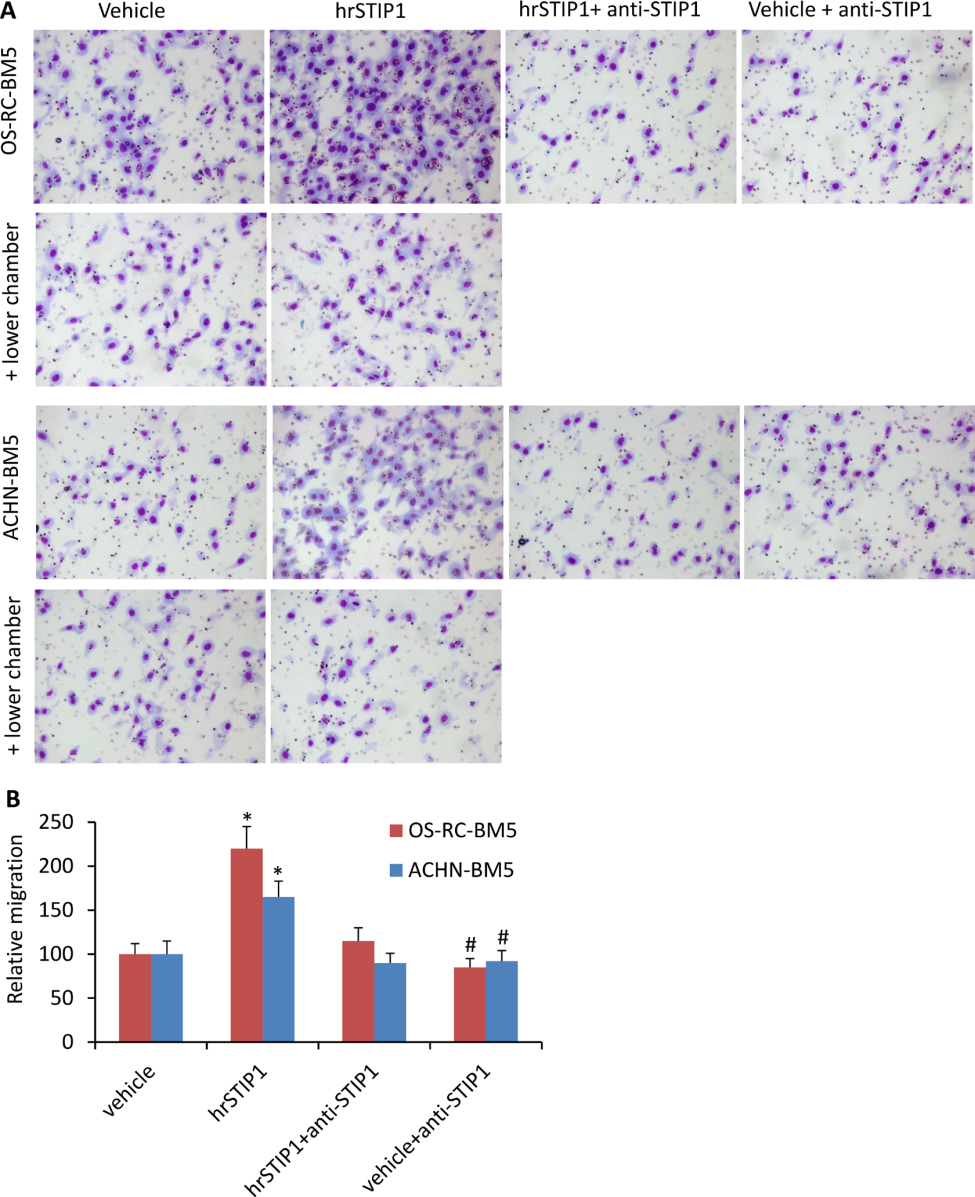
STIP1 promotes tumor cell migration/invasion (**A**) Representative images of the Transwell membranes with tumor cells migrated to the counter side of the chamber. Note, cell migration was not affected when hrSTIP1 was added into the lower chamber (+ lower chamber). (**B**) Quantification of the migration analysis with three repeats. *p < 0.05, *vs* vehicle; ^#^*p <* 0.05, vs hrSTIP1+anti-STIP1.

The Boyden chamber migration and invasion assay were used to investigate the effects of STIP1 on the RCC tumor cells. The OS-RC-2-BM5 and ACHN-BM5 cells were serum-starved overnight and pre-treated with hrSTIP1 for 12 h before seeding them in the upper chamber. Sixteen hours later, we found that significantly more cells had penetrated to the counter-side of the upper chamber in the hrSTIP1-treated groups than in the vehicle-treated groups (Figure 5). Co-treatment with the anti-STIP1 neutralizing antibody not only abrogated these effects but also reduced the naive migration/invasion of the OS-RC-2-BM5 cells without STIP1 addition (Figure 5A, 5B). However, when the hrSTIP1 was applied into the lower chamber, we did not observe any significant changes in the number of penetrating cells (Figure 5A, + lower chamber). These results clearly demonstrated that both endogenous and exogenous STIP1 promoted RCC tumor cell migration and invasion, and direct interaction with tumor cells is indispensable to mediate STIP1's effect.

**Figure 6:**
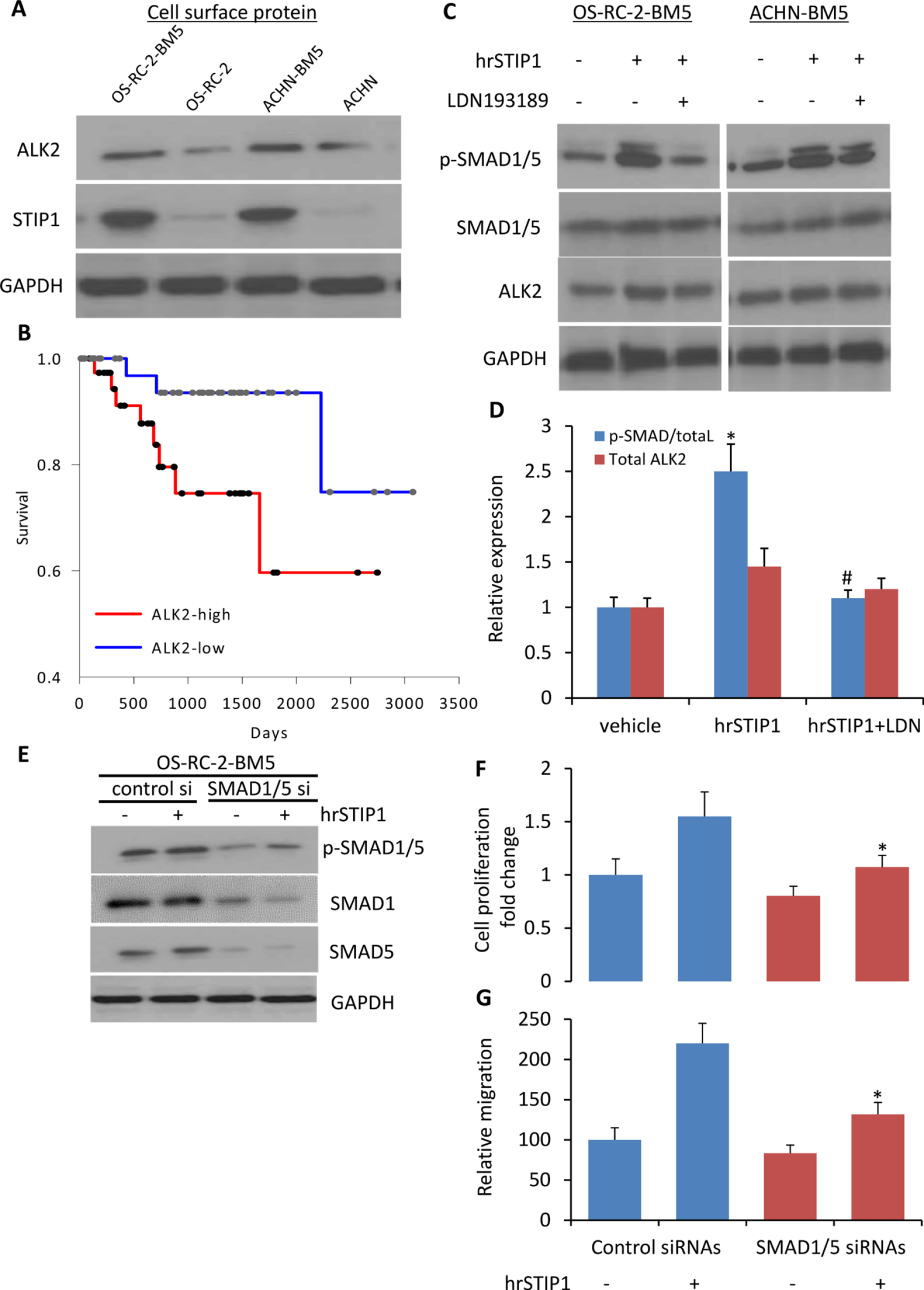
Activation of STIP1-ALK2-SMAD1/5 signaling in bone metastatic RCC tumor cells (**A**) ALK2 expression was examined in the cell surface protein of indicated cell lines. (**B**) The Kaplan-Meier curve for overall survival of the TCGA RCC cohort (*n* = 88) on the basis of ALK2 mRNA level. Patient was determined as ALK2-high or –low group when the ALK2 expression value was above or below the mean value in the dataset. The survival distributions were estimated by the Kaplan-Meier method, and the significance of differences between survival rates was ascertained using the log-rank test. (**C**) Expressions of p-SMAD1/5, SMAD1/5, ALK2 in the total cell lysate from indicated cell lines upon hrSTIP1 and/or LDN193189, or corresponding vehicle treatment. (**D**) Quantification of the western blot analysis (C) with three repeats. Expression of p-SMAD1/5 was normalized to the level of total SMAD1/5, and ALK2 expression was normalized to the level of GAPDH. *p < 0.05, *vs* vehicle; ^#^*p <* 0.05, vs hrSTIP1. (**E**) Knockdown of endogenous SMAD1 and SMAD5 by siRNA decreased the stimulation of pSMAD1/5 protein levels by treatment with 500 nM hrSTIP1. F-G. Suppression of endogenous SMAD1/5 by siRNAs decreased the 96-h proliferation (**F**) and 24-h migration (**G**) of OS-RC-2-BM5 cells under treatment with 500 nM hrSTIP1. Results shown are the mean ± SE from three independent experiments. *p < 0.05, *vs* control siRNAs + hrSTIP1.

Although high expression of STIP1 has been reported in various cancers, STIP1 signal transduction is much less studied. One report showed that STIP1 induced ovarian cancer cell proliferation through binding with the activin A receptor, type II-like kinase 2 (ALK2) [15]. In our study, we found that the ALK2 protein was highly expressed on the cell membrane of OS-RC-2-BM5 and ACHN-BM5 cells (Figure 6A), and the high expression of *ALK2* mRNA correlated with shorter overall survival in the 88-patient TCGA Renal cohort analysis (*P* = 0.041, log-rank test. Figure 6B). Furthermore, downstream to the ALK2, activation of SMAD1/5 was detected in the cells upon the hrSTIP1 treatment, and the ALK2 inhibitor LDN193189 completely suppressed the hrSTIP1-induced SMAD1/5 activation (Figure 6C, 6D). To further confirm the involvement of SMAD1/5 in the STIP1-induced proliferation and migration of bone-seeking RCC cells, knockdown of endogenous SMAD1 and SMAD5 by siRNAs was conducted in the OS-RC-2-BM5 cell line. The results showed that suppression of SMAD1/5 attenuated hrSTIP1 stimulation of cell proliferation and migration (Figure 6E–6G). SMAD1 and 5 are the canonical bone morphogenetic protein effectors, and the bone morphogenetic protein (BMP) pathways are involved in various cell functions, including cell proliferation and migration [16]. Although SMAD1/5 knockdown diminished the proliferation and migration of OS-RC-2-BM5 cells (∼20%), the knockdown cells exhibited decreased sensitivity to hrSTIP1 addition compared to the control cells in terms of proliferation (55% vs. 34%) and migration (2.2-fold vs. 1.58-fold), implicating the dependence of STIP1 on SAMD1/5 signaling. These results support that the autocrine STIP1-ALK2-SMAD1/5 in the bone metastatic RCC tumor cells is the dominant pathway mediating the enhanced cell proliferation and migration/invasion.

### STIP1 promotes osteoclast differentiation through the PrPc-ERK1/2 but not ALK2-SMAD1/5 pathway

**Figure 7:**
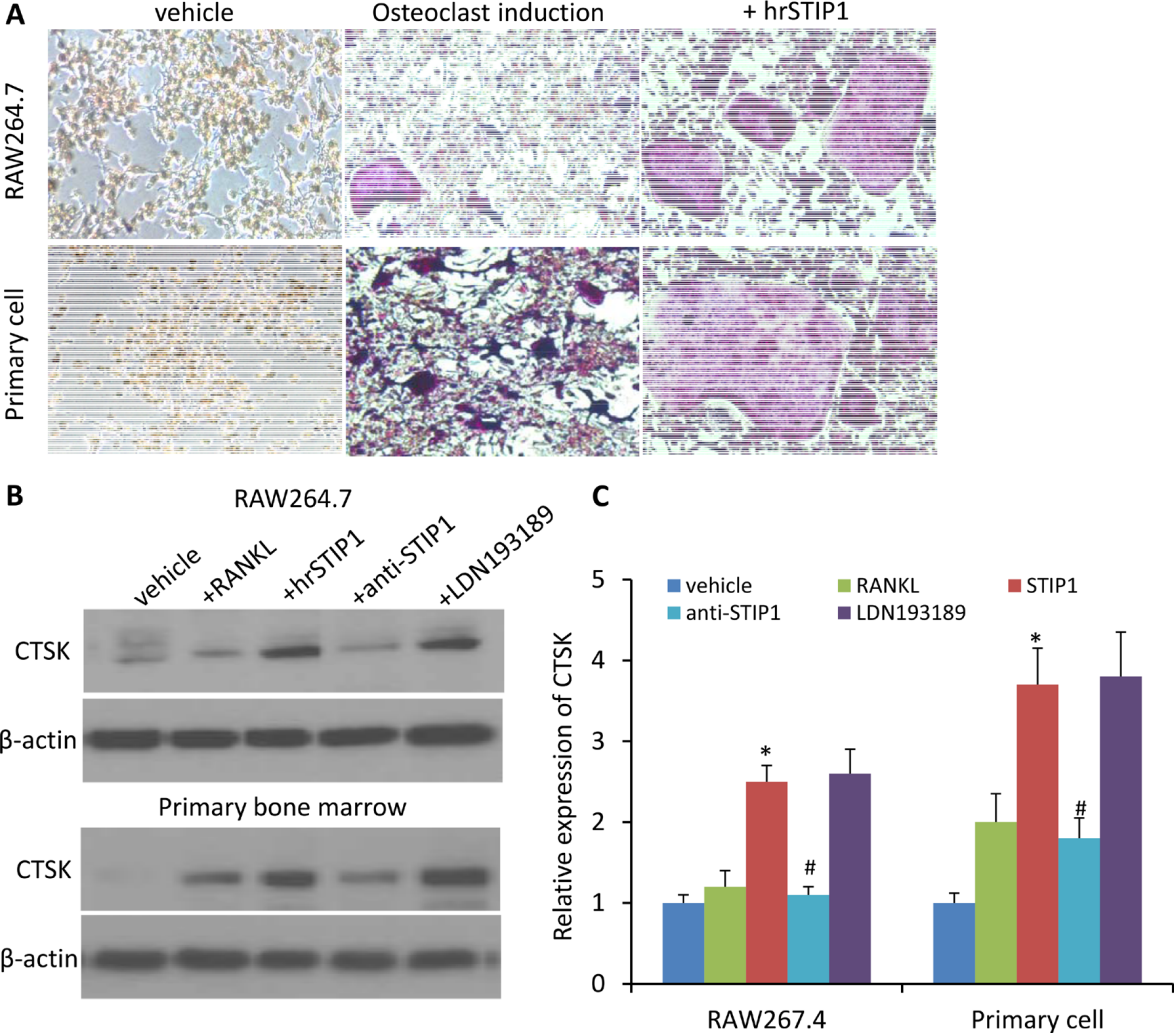
STIP1 promotes osteoclast differentiation (**A**) TRAP staining of osteoclasts induced in RAW264.7 cell line and primary bone marrow cells upon treatment with hrSTIP1 (500 nM). (**B**) Expressions of CTSK in the total cell lysate from indicated treatment in both RAW264.7 cell line and primary bone marrow cells. (**C**) Quantification of the western blot analysis (B) with three repeats. Expression of CTSK was normalized to the level of β-actin. *p < 0.05, *vs* vehicle; ^#^*p <* 0.05, vs hrSTIP1. Western blots images shown are cropped to show the protein of interest, and all blots were performed under the same experimental conditions.

ALK2 belongs to the BMP receptor family, which is involved in the skeletal development and fracture healing processes. To explore whether STIP1 has paracrine effects on osteocytes through ALK2 to promote osteolytic bone metastasis, we first conducted an osteoclast-like differentiation experiment on the murine preosteoclast cell line RAW264, treated for three days with recombinant mouse RANKL. The phenotype of RANKL-treated RAW264.7 cells was osteoclast-like as determined by tartrate-resistant acid phosphatase (TRAP) activity and TRAP staining [17]. When hrSTIP1 (500 nM) was co-administered with 100 ng/ml RANKL to the RAW264.7 cells, significantly more differentiated osteoclasts were observed after quantitatively analyzing the TRAP+ cells in the whole well-montage images (Figure 7A). In addition, immunoblotting for cathepsin K (CTSK), the key enzyme secreted by osteoclasts to degrade collagen and other matrix proteins during bone resorption, revealed a 2-fold elevation in the hrSTIP1-treated cells, compared with the RANKL only treated cells (Figure 7B, 7C). Secondly, we harvested the bone marrow cells from femur of C57BL/6 mice for primary osteoclast differentiation experiment. Similar to what we observed on the RAW264.7 cell line, treatment of hrSTIP1 enhanced TRAP+ staining and CTSK expression in the cells with the presence of mouse macrophage colony-stimulating factor (M-CSF) (50 ng/mL) and RANKL (50 ng/mL) for seven days (Figure 7A–7C). Furthermore, the anti-STIP1 neutralizing antibody significantly abrogated the effects of STIP1 on osteoclast-related CTSK in both cell line and primary cell settings (Figure 7B, 7C). However, the specific ALK2 inhibitor LDN193189 showed minimal but not significant interruption to STIP1's effects (Figure 7B, 7C), which may suggest the existence of other pathways in mediating the induction of osteoclast differentiation by STIP1. In addition, from the Oncomine database analysis, *ALK2* expresses at a very low level in normal adult human bone marrow tissue (Supplementary Figure 1A), further helping us to exclude the involvement of the ALK2 pathway in mediating the tumor-osteoclast interactions.

**Figure 8:**
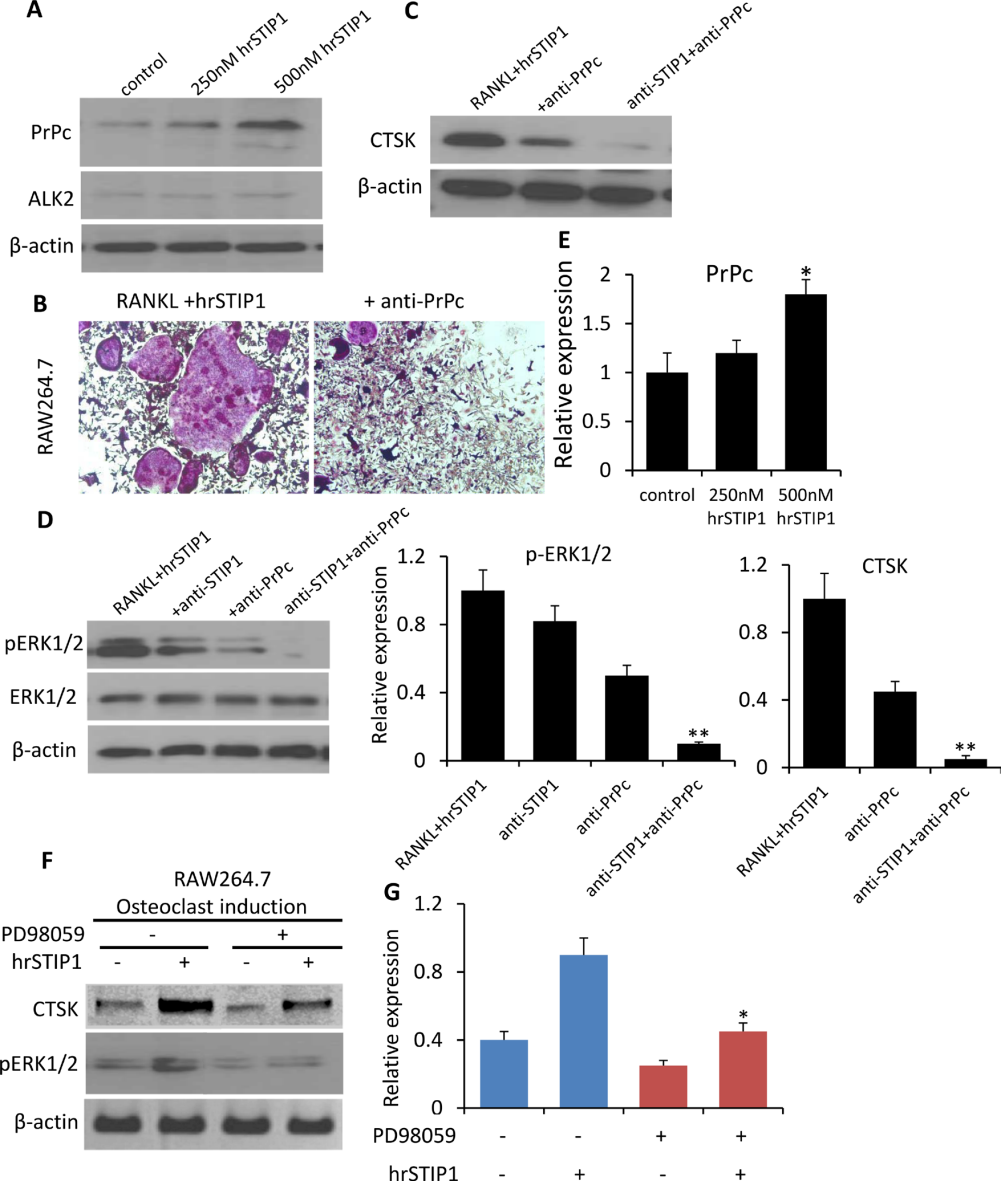
Activation of STIP1-PrPc-ERK1/2 signaling in osteoclast differentiation (**A**) Expression of PrPc and ALK2 in the total cell lysate from hrSTIP1 treated-RAW264.7 cells. (**B**) TRAP staining of osteoclasts induced in RAW264.7 cell line upon treatment with hrSTIP1 (500 nM) or hrSTIP1+anti-PrPc (10 μg/ml) for 48 hours. (**C**) Expression of CTSK in the total cell lysate from indicated treatment in RAW264.7 cells. (**D**) Expression of p-ERK1/2 and total ERK1/2 in the total cell lysate from indicated treatment in RAW264.7 cells. (**E**) Quantification of the western blot analysis (A, C, D) with three repeats. Expression of PrPc and CTSK were normalized to the level of β-actin, and expression of pERK1/2 was normalized to total ERK1/2. *p < 0.05, *vs* control; **p < 0.01, vs RANKL+hrATIP1. (**F**) Suppression the activation of endogenous ERK1/2 signaling by pre-treatment with 15 μM PD98059 for 2-h inhibited the hrSTIP1-induced CTSK protein expression in RAW264.7 cells. (**G**) Quantification of the western blot analysis of CTSK expression with three repeats. *p < 0.05, *vs* PD98059 -/hrSTIP1+.

STIP1 has been identified as a cell surface ligand for the cellular prion protein PrPc [18]. Several studies have linked PrPc to human cancers [19]. Although PrPc mRNA is expressed at a low level in normal adult human bone marrow in the Oncomine database analysis (Supplementary Figure 1B), we detected a dose-dependent induction of PrPc expression in the RAW264.7 cells by hrSTIP1 (Figure 8A, 8E), and the anti-PrPc antibody (10 μg/mL) was able to remarkably block the STIP1-induced RAW264.7 osteoclast differentiation (Figure 8B). A combination of anti-STIP1 and anti-PrPc antibodies was able to inhibit the CTSK expression almost completely (Figure 8C, 8E). The downstream ERK1/2 activation induced by hrSTIP1 was also inhibited upon the anti-STIP1 and/or anti-PrPc antibody treatment (Figure 8D, 8E). In addition, suppressing the activation of endogenous ERK1/2 signaling by pre-treatment with the specific MEK inhibitor PD98059 inhibited the hrSTIP1-induced CTSK protein expression in differentiating RAW264.7 cells (Figure 8F, 8G). Pre-treating the cells with 20 μM PD98059 for 2 hours didn't cause obvious cytotoxicity (data not shown)[20], but the suppression of ERK1/2 led to a moderate inhibition on osteoclast differentiation of the RAW264.7 cells, which is in concordance with previous reports [21]. With the addition of hrSTIP1 to the culture system, the PD98059 pretreated cells showed significantly less response in expressing CTSK than the cells without pretreatment (1.8-fold vs 2.25-fold) (Figure 8G), implying the dependence of STIP1's effect on ERK1/2 activity. Only partial reduction but not complete abolishment of the hrSTIP1's stimulation on osteoclast differentiation in RAW264.7 cells certainly suggests the existence of a downstream molecular mechanism other than ERK1/2, but these results indicated that the STIP1-PrPc-ERK1/2 pathway was involved in the overexpression of osteolytic CTSK.

## DISCUSSION

To the best of our knowledge, this study is the first to report the autocrine and paracrine STIP1 signaling in bone metastasis of RCC. The autocrine STIP1-ALK2-SMAD1/5 pathway promotes tumor cell survival, and the paracrine STIP1-PrPc-ERK1/2 pathway aggravates osteolysis. The interplay between these two pathways may represent a vicious cycle between tumor cell and bone niche, contributing to the early osteolytic progression—a leading feature of RCC bone metastasis.

STIP1 was first identified as a renal carcinoma antigen NY-REN-11, a tumor antigen recognized by the humoral immune system [22]. It is also known as transformation-sensitive protein IEF SSP 3521, a protein overexpressed in transformed cells, and HSP70/HSP90-organizing protein (HOP), a protein involved in heat shock response. In cancers, STIP1 can be translocated to cell surface or secreted out of the cell by ovarian cancer [14, 15] and glioblastoma cells [23], and stimulates cancer cell proliferation in an autocrine manner. In our previous study, we obtained the enriched bone-seeking RCC cell line OS-RC-2-BM5 from *in vivo* selection [12], and here we detected a remarkably high expression of STIP1 on the cell surface membrane which also led to the secretion of STIP1 protein into the culture medium, indicating a possible autocrine effect of STIP1 on the bone-seeking tumor cell itself. In animal models, OS-RC-2-BM5 cells induced bone metastasis in 50% of injected mice starting from as early as 3 weeks after injection [12]. Knockdown of STIP1 by specific shRNAs in the OS-RC-2-BM5 cells led to a more than 50% inhibition of cell proliferation, and patients with low STIP1 expression also showed less Ki67-positive proliferating tumor cells in their bone metastatic lesions, suggesting the pro-proliferation role of STIP1 on the bone-seeking cells. The effective blockade of hrSTIP1-stimulated cell proliferation and migration of the bone-seeking cells elicited by anti-STIP1 antibody further corroborated the role of STIP1 on tumor cells. STIP1 has been reported to be up-regulated in various types of cancer [23–26]. The STIP1 gene is located at 11q13, and copy number gain of this region has often been shown in cancers and is linked to poor prognosis [27–30]. STIP1 represents an attractive potential target for cancer treatment. For example, a compound that blocks HSP90 interaction with STIP1 impairs the HSP90-dependent folding pathway and is toxic to breast cancer cells [31]. Moreover, an *in vitro* study by Horibe et al. has reported that an anti-TPR peptide that blocks the interaction of HSP90 with the TPR domain of STIP1 is induces cell death in lung, renal, prostate, pancreatic, and gastric cancer cell lines [32]. STIP1 is well studied as HOP, and the heat shock response signaling are extensively involved in tumor genesis and cancer progression [33]. Although we were more focused on verifying that the cell membrane ALK2 was the major receptor in transducing the STIP1 ligand signal in this study, the HSP90-STIP1 mechanism may also contribute to the outgrowth of the bone metastatic RCC tumor cells, and our study could not exclude this. Specifically, we observed a significant increase of the overall expression of STIP1 in the total cell lysate, including the intracellular STIP1, and this fraction of STIP1 will most likely interact with HSP90. Overall, our results suggest that a combination strategy to inhibit both the extracellular STIP1-ALK2 signaling and intracellular STIP1-HSP90 signaling would be ideal to thoroughly block STIP1-induced tumor growth in RCC bone metastasis.

Bone metastases from RCC are almost always highly destructive and purely osteolytic. Most *in vivo* studies indicate that osteolysis is caused by osteoclast stimulation [34]. In the OS-RC-2-BM5 xenograft mouse model, reduced trabeculae and thin bone cortex that are mostly accompanied with osteolytic destruction were commonly observed in long bones [12], and TRAP staining-positive osteoclasts were obviously more examined surrounding the tumor lesions than the non-tumor areas (Supplementary Figure 2), indicating a osteoclast resorption activity. Osteoclasts are derived from precursor mononuclear phagocytic cells in the bone marrow, and a plethora of systemic and locally acting factors can impact osteoclast maturation [34]. In the *in vitro* mouse preosteoclast cell line and primary bone marrow cells, our data showed that the STIP1-PrPc-ERK1/2 pathway was involved in promoting the osteoclast differentiation and expression of osteolytic CTSK. A wealth of data have exemplified that PrPc is expressed at the cell surface of distinct types of hematopoietic stem cells and regulates their self-renewal and differentiation potential [35], and STIP1 has been shown in binding with PrPc to sustain the self-renewal of neural progenitor/stem cells [36]. Our data is the first to show that PrPc potentiates osteoclast differentiation. Although a direct binding assay was not performed to confirm the interaction between STIP1 and PrPc in the osteoclast precursor cells, the results from several functional studies were supportive to our conclusion, i.e., 1) a dose-dependent induction of PrPc expression by hrSTIP1; 2) the anti-PrPc antibody impeded the hrSTIP1-induced osteoclast differentiation; and 3) the combination of anti-STIP1 and anti-PrPc antibodies inhibited the CTSK expression significantly. *In vivo*, several cancer-associated conditions, including hypoxia and oxidative or endoplasmic reticulum stresses can activate PrPc transcription [37, 38], which also been shown to increase osteoclast activity [39, 40]. Thus, the STIP1-PrPc signaling in osteolysis might be augmented and an *in vivo* verification in the bone metastasis RCC animal model or patient specimens would be needed.

During cancers progression, reciprocal molecular exchanges between tumor cells and the surrounding stroma promote malignancy, stimulate tumor growth and induce stromal remodeling. Our study indicates that STIP1 is such a molecule in mediating the interplay between tumor cells and bone niche in bone metastasis from RCC. The bone-seeking RCC cells have an intrinsic high expression and secretion of STIP1, which promotes tumor cell proliferation and osteoclast maturation. It is well known that bone matrix is a rich source of stored growth factors such as TGF-β and insulin-like growth factor (IGF). Once released from degraded bone matrix, such growth factors may further accelerate tumor growth, which can expand within the lysed area. Our results also show that ALK2 is highly expressed on the surface of bone-seeking tumor cells. ALK2 is a TGF-β receptor superfamily member and is able to transduce the TGF-β signaling in promoting tumor outgrowth [41]. The growth of tumor cells will further increase the release of osteolytic mediators, STIP1 and others, such as PTHrP [34]. The initial release of osteolytic mediators by tumor cells leads to bone degradation, release of growth factors from degraded bone, increased tumor cell proliferation, and finally further release of osteolytic mediators, which forms a tumor-enhancing feedback cycle. It has been noted that the physical properties of bone matrix, including low oxygen content, acidic pH, plus growth factors, create an environment favorable for tumor growth [42], and also activate the PrPc transcription to transduce the STIP1 stimulation for osteoclast differentiation [37, 38]. Thus, the tumor STIP1-ALK2 signaling and osteoclast STIP1-PrPc signaling aggravate the vicious cycle in osteolytic bone metastasis from RCC.

Bone metastasis occurs in 35% to 40% of advanced RCC cases, and causes significant morbidity through skeletal related events [43]. Until recently, a limited number of systemic treatments were available for advanced RCC and few new candidates have emerged, including compounds targeting the vascular endothelial growth factor (VEGF) axis or mTOR. However, these drugs’ effects on bone metastases are far less clear. Data suggests that the third-generation bisphosphonate, zoledronic acid, benefits patients with bone metastases from advanced RCC, but the data was gathered prior to the targeted therapy era; therefore, there is some uncertainty about the role in patients on modern RCC therapies. If corroborated by further *in vivo* studies, our data suggest that STIP1 may serve as a promising target for a new therapeutic strategy in bone metastases from advanced RCC.

## MATERIALS AND METHODS

### Cell lines and osteoclast differentiation

The bone-seeking OS-RC-2-BM5 and ACHN-BM5 cell lines were previously described [11, 12]. Their parental cell lines, OS-RC-2 and ACHN, as well as the mouse monocyte-macrophage RAW264.7 cell line, were purchased from China Center for Type Culture Collection (CCTCC), Wuhan, China. Cell line characterization or authentication was performed with short-tandem repeat profiling and cells were passaged in our laboratory for less than six months after receipt.

For osteoclast-like differentiation experiments, RAW264.7 cells were cultured in α-MEM supplemented with 10% (v/v) FBS, 1% (v/v) penicillin-streptomycin solution, and 10mM HEPES solution and incubated at 37°C in 5% CO_2_ humidified air. The medium was changed every 3 days. RAW264.7 cells were seeded at 4 × 10^5^ cells/cm^2^ in 6- or 12-well culture dishes and incubated overnight prior to treatment with 100 ng/ml RANKL (Boster Biotechnology, Wuhan, China) for 3 days to induce a phenotype similar to osteoclasts [17]. For bone osteoclast differentiation, bone marrow cells were harvested from femurs of 8 week-old male C57BL/6 mice (Experimental Animal Center of Huazhong University of Science and Technology, Wuhan, China) under aseptic conditions and pooled. Cells were cultured overnight in 10% FBS then seeded in 24-well plate at 1 × 10^5^ cells/well and cultured with 50 ng/mL of mouse M-CSF (Boster Biotechnology, Wuhan, China) for 3 days, and followed with M-CSF and 50 ng/mL RANKL for 7 days. Media was changed every 3 days. 500 nM recombinant human STIP1 (hrSTIP1) (Boster Biotechnology, Wuhan, China), 800 nM anti-STIP1 antibody (Abcam, Cambridge, USA), 10 μg/mL anti-PrPc antibody (Abcam, Cambridge, USA), and 20 μM PD98059 (Boster Biotechnology, Wuhan, China) were used to treat the tumor cells for certain assays.

### Cell proliferation assay and cell cycle analysis

We used the MTT assay to determine cell proliferation. Cell cycle analysis was measured by flow cytometry. The experimental procedures have been previously described [11, 12]. All assays were repeated three times.

### Cell migration and invasion assay

Tumor cells treated with either hrSTIP1 (500 nM) or the vehicle alone, were serum-starved overnight and pre-treated with hrSTIP1 for 12 h. Cells were then plated into the upper chamber of a Transwell (24-well, 8-μm pore size; Deyi Biological Technology Co., Wuhan, China). The lower chamber was filled with 800 μL of DMEM/F12 and 0.5 μg/mL of fibronectin (Boster Biotechnology, Wuhan, China). After a 16 hour incubation, cells that had migrated through pores and adhered to the lower membrane were stained with 1% crystal violet in 2% ethanol for 20 min (Deyi Biological Technology co., Wuhan, China). The membrane was imaged using an IX61 Olympus microscope (Olympus Optical, Tokyo, Japan), and the number of viable cells that had traversed the filter was counted by the Slidebook software. 800 nM anti-STIP1 antibody was added to cancer cells previously exposed to 400 nM hrSTIP1 for 6 hours before the Transwell assay was performed. All assays were repeated at least three times.

### Stable shRNA knockdown

Knockdown of STIP1 was achieved by a validated hairpin STIP1 shRNA Lentiviral Particle Gene Silencer kit (Santa Cruz Biotechnology, Texas, USA). To knock down the expression of SMAD1/5, two siRNA pools were combined, 50 nM of Smad1 siRNA and 25 nM of Smad5 siRNA were transfected. Plasmid transfections were performed using Lipofectamine2000 reagent (Invitrogen) according to the manufacturer's protocol.

### TRAP staining

TRAP staining kit (Sigma-Aldrich, St. Louis, MO USA) was used to quantify the number of mature osteoclasts. Briefly, cells were fixed with 10% buffered formalin for 2 min. After rinsing in deionized water, cells were put into TRAP staining solution for 1 h according to the manufacturer's protocol. Stained cells were then imaged on an Olympus IX71 microscope (Olympus Optical, Tokyo, Japan).

### Protein detection in cell culture supernatant and outer cell surface

RCC cancer cells were cultured overnight in serum-free Opti-MEM (Invitrogen, Carlsbad, CA USA). After centrifugation at 1600 rpm for 10 min to remove cell debris, 45 μl of culture supernatant was analyzed by Western blot for detecting STIP1. GAPDH was used as a control to detect whether the intracellular protein had leaked into the culture supernatant. Pierce Cell Surface Protein Isolation Kit (Thermo Scientific, Carlsbad, CA USA) was used to isolate cell membranous proteins for detecting STIP1. The moderate cytosolic level of HSP90 was used as a marker for measuring intercellular protein contamination in surface protein isolates.

### Western blot

Cell or tissue lysates and immunoblot analysis were performed as described previously [11, 12]. Densitometric analysis was conducted using ImageJ software. The primary antibodies used include: anti-STIP1 (1:200, Boster Biotechnology, Wuhan, China), anti-ALK2 (1:400, Boster Biotechnology, Wuhan, China), anti-SMAD1/5 (1:200, Boster Biotechnology, Wuhan, China), anti-CTSK (1:500, Boster Biotechnology, Wuhan, China), anti-ERK1/2 (1:500, Boster Biotechnology, Wuhan, China), anti-Hsp90 (1:500, Boster Biotechnology, Wuhan, China), anti-Ki67 (1:500, Boster Biotechnology, Wuhan, China) and anti-GAPDH (1:500, Boster Biotechnology, Wuhan, China).

### Patient specimens and immunohistochemistry

Tissue biopsy or surgically removed fresh primary RCC samples (*n* = 7) and bone metastasis samples (*n* = 12) were used to quantify the expression of the STIP1 protein by Western blot analysis. Ten pairs of matched primary RCC and bone metastasis paraffin-embedded tissues were used to examine the immunoreactivity of the STIP1 protein. All samples were obtained from Tongji Hospital between Mar 2001 and Dec 2015; the consent forms and the studies were approved by the IRB committee at Tongji Medical College, Huazhong University of Science and Technology. All the primary RCC tumors were clarified as clear cell histotype by the pathology department.

The formalin-fixed, paraffin-embedded tissue sections were stained with a primary mouse anti-human STIP1 monoclonal antibody (1:200, Boster Biotechnology, Wuhan, China) with the Ventana Basic DAB (3,3-diaminobenzidine) Detection kit (Boster Biotechnology, Wuhan, China). Slides were evaluated independently by two pathologists who were blinded to the clinicopathological data and the patients’ identities. The overall immunohistochemical score (histoscore) was calculated as the percentage of positive tumor cells (0−100%) multiplied by staining intensity (0 = negative, 1 = weak, 2 = moderate, 3 = strong). Therefore, the total histoscore ranged from 0 to 300. Anti-STIP1 antibody specificity was validated by blocking the antibodies with 500 nM of hrSTIP1 during the incubation step with anti-STIP1.

### Patient microarray and Oncomine gene expression data analysis

TCGA Renal cohort (*n* = 88) in which microarray and clinical data are publicly available were used for the correlation analysis between STIP1 or ALK2 and clinical outcomes. Relative levels of ALK2 mRNA expression in normal human tissues were obtained by Oncomine database analysis (http://www.oncomine.com). The data was log 2-transformed, with the media set to zero and s.d. set to one.

### Statistical analysis

Data are expressed as means ± SEM. To compare groups, we used the Student's two-tailed *t* test or the Mann-Whitney rank sum test. To assess correlations, we calculated the Spearman's rank correlation coefficient. To compare survival, we used log-rank test. *P <* 0.05 was regarded as statistically significant. We performed all calculations with SigmaPlot statistical software (version 11.2; Systat Software Inc. Chicago, IL).

## SUPPLEMENTARY MATERIALS AND FIGURES


