# Supplementary material for: Autocrine and paracrine STIP1 signaling promote osteolytic bone metastasis in renal cell carcinoma
Source: Oncotarget. 2017 Feb 9;8(10):17012–26. doi: 10.18632/oncotarget.15222 (PMC5370018; doi:10.18632/oncotarget.15222)
Supplement: Supplementary file 1 [file oncotarget-08-17012-s001.pdf]

Autocrine and paracrine STIP1 signaling promote osteolytic bone metastasis in renal cell carcinoma

Supplementary Materials

A

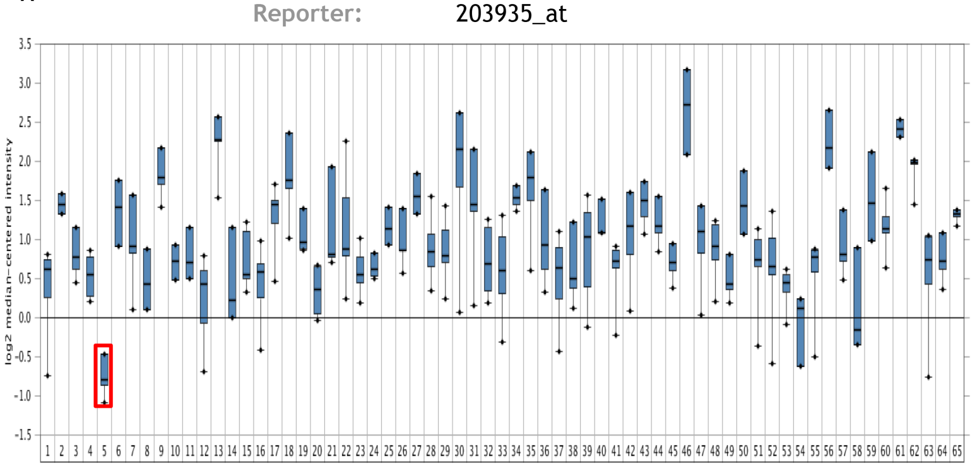

Legend

1. Accumbens Nucleus (9) 2. Adipose Tissue (3) 3. Adrenal Cortex (4) 4. Amygdala (8) 5. Bone Marrow (5) 6. Bronchus (3) 7. Buccal Mucosa (4) 8. Gastric Cardia (3) 9. Cardiac Atrium (4) 10. Cardiac Ventricle (3) 11. Cecum (3) 12. Cerebellum (9) 13. Cervix Uteri (4) 14. Coronary Artery (3) 15. Corpus Callosum (9) 16. Cerebral Cortex (9) 17. Dorsal Root Ganglion (8) 18. Endometrium (4) 19. Esophagus (4) 20. Frontal Lobe (9) 21. Fundus of the Stomach (4) 22. Hippocampus (9) 23. Hypothalamus (8) 24. Liver (4) 25. Lung (3) 26. Lymph Node (4) 27. Mammary Gland (3) 28. Medulla Oblongata (9) 29. Mesencephalon (9) 30. Myometrium (5) 31. Nipple (4) 32. Nodose Ganglion (8) 33. Occipital Lobe (8) 34. Omental Adipose Tissue (4) 35. Ovary (4) 36. Papilla of the Tongue (4) 37. Parietal Lobe (9) 38. Pharyngeal Mucosa (4) 39. Pituitary Gland (8) 40. Prostate Gland (3) 41. Putamen (9) 42. Pylorus (4) 43. Renal Cortex (4) 44. Renal Medulla (4) 45. Salivary Gland (4) 46. Saphenous Vein (3) 47. Skeletal Muscle Tissue (5) 48. Spinal Cord (8) 49. Spleen (4) 50. Subcutaneous Adipose Tissue (3) 51. Substantia Nigra (8) 52. Subthalamic Nucleus (8) 53. Temporal Lobe (8) 54. Testis (3) 55. Thalamus (8) 56. Thyroid Gland (4) 57. Tongue (4) 58. Tonsil (3) 59. Trachea (3) 60. Trigeminal Ganglion (8) 61. Urethra (3) 62. Vagina (4) 63. Ventral Tegmentum (8) 64. Vestibular Nucleus (7) 65. Vulva (4)

Roth Normal

Neurogenetics 2006/05/01	353 samples
mRNA	19,574 measured genes
Human Genome U133 Plus 2.0 Array	

B

Reporter: 201300_at

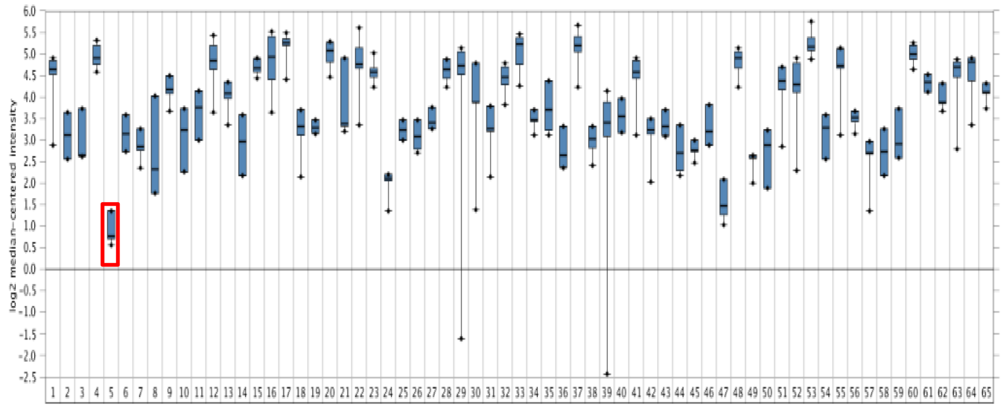

Legend

1. Accumbens Nucleus (9) 2. Adipose Tissue (3) 3. Adrenal Cortex (4) 4. Amygdala (8) 5. Bone Marrow (5) 6. Bronchus (3) 7. Buccal Mucosa (4) 8. Gastric Cardia (3) 9. Cardiac Atrium (4) 10. Cardiac Ventricle (3) 11. Cecum (3) 12. Cerebellum (9) 13. Cervix Uteri (4) 14. Coronary Artery (3) 15. Corpus Callosum (9) 16. Cerebral Cortex (9) 17. Dorsal Root Ganglion (8) 18. Endometrium (4) 19. Esophagus (4) 20. Frontal Lobe (9) 21. Fundus of the Stomach (4) 22. Hippocampus (9) 23. Hypothalamus (8) 24. Liver (4) 25. Lung (3) 26. Lymph Node (4) 27. Mammary Gland (3) 28. Medulla Oblongata (9) 29. Mesencephalon (9) 30. Myometrium (5) 31. Nipple (4) 32. Nodose Ganglion (8) 33. Occipital Lobe (8) 34. Omental Adipose Tissue (4) 35. Ovary (4) 36. Papilla of the Tongue (4) 37. Parietal Lobe (9) 38. Pharyngeal Mucosa (4) 39. Pituitary Gland (8) 40. Prostate Gland (3) 41. Putamen (9) 42. Pylorus (4) 43. Renal Cortex (4) 44. Renal Medulla (4) 45. Salivary Gland (4) 46. Saphenous Vein (3) 47. Skeletal Muscle Tissue (5) 48. Spinal Cord (8) 49. Spleen (4) 50. Subcutaneous Adipose Tissue (3) 51. Substantia Nigra (8) 52. Subthalamic Nucleus (8) 53. Temporal Lobe (8) 54. Testis (3) 55. Thalamus (8) 56. Thyroid Gland (4) 57. Tongue (4) 58. Tonsil (3) 59. Trachea (3) 60. Trigeminal Ganglion (8) 61. Urethra (3) 62. Vagina (4) 63. Ventral Tegmentum (8) 64. Vestibular Nucleus (7) 65. Vulva (4)

Roth Normal

Neurogenetics 2006/05/01	353 samples
mRNA	19,574 measured genes
Human Genome U133 Plus 2.0 Array	

Supplementary Figure 1: Oncomine database analysis of ALK2 (**A**) and PrPc (**B**) mRNA expression in normal human tissue (Neurogenetics 2006 7:67−80).

A **B**

TRAP staining

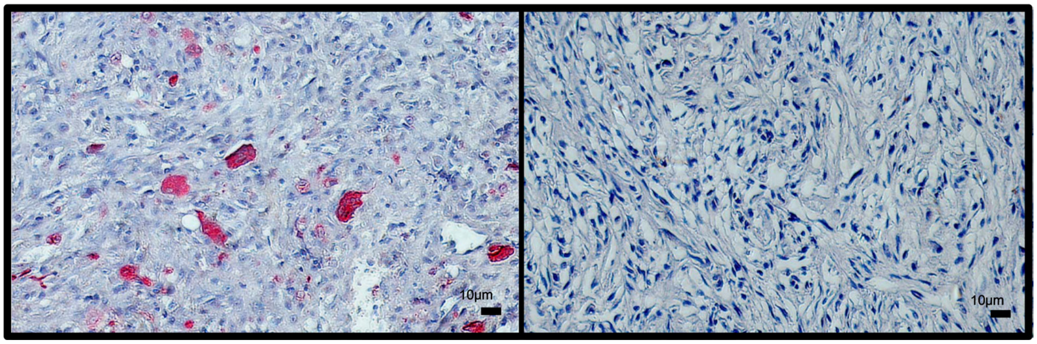

Supplementary Figure 2: TRAP staning in OS-RC-2-BM5 (**A**) and non-tumor area (**B**). A: trap staining in bone lesion from mouse model with OS-RC-2-BM5 B trap staining in peritumorous connective tissue from mouse model.
